# Experimental investigation of the dynamic cavitation behavior and wall static pressure characteristics through convergence-divergence venturis with various divergence angles

**DOI:** 10.1038/s41598-020-68317-3

**Published:** 2020-08-25

**Authors:** Yinan Liu, Hua Fan, Di Wu, Haijun Chen, Kuolei Feng, Chaocheng Zhao, Dongyin Wu

**Affiliations:** School of Energy and Power Engineering, Xi’an Jiaotong University, Xi’an, 710049 China

**Keywords:** Physics, Fluid dynamics

## Abstract

As a highly efficient and energy-saving cavitation method, Venturi cavitation is widely used in many industrial fields. This study synchronously investigated the cavity behavior and its corresponding wall static pressure characteristics in Venturi channels with various divergence angles to research the role of the divergence angle in cavity shape and the wall static pressure oscillation. Five rectangular Venturi channels with different divergence angles (4°, 6°, 8°, 10°, and 12°) were tested at the cavitation number (0.3–1.0). Based on the dynamic behaviour of gas–liquid interface, three cavity shedding types were identified: front shedding (I), central shedding (II) and tail shedding (III). A modified correlation for predicting average cavity length was proposed with the consideration of the effect of the divergence angle. Combined with the wall static pressure characteristics, as the divergence angle increased, the wall static pressure fluctuation in the Venturi became more intense. According to the wall static pressure oscillation characteristics, for the larger divergence angles (*θ* = 6°, 8°, 10° and 12°), the wall static pressure oscillation frequency was the same as the cavity shedding frequency and increased with the increase of the divergence angle. For smaller divergence angle (*θ* = 4°), no definite periodicity in pressure oscillation frequency could be observed.

## Introduction

Cavitation is a physical phenomenon in which a violent phase change is caused by the decrease of the partial pressure of liquid below the vapour saturated pressure. Cavitation in industrial processes often has adverse effects. For instance, cavitation not only causes erosion, noise and vibration in a variety of fluid machinery, such as impeller blades, pumps and turbines, but also decreases the efficiency of the Venturi mixer^1–5^. Cavitation also has positive effects in the aerospace, biological, and chemical industries, among others. It can be used for engine flow control, sewage purification and chemical reaction rate increase^6–9^. The adverse effects and positive effects of cavitation are manifested in various cavitation processes. The cavity length and the flow field pressure are important indicators for characterizing cavitation processes, which mainly reflect cavitation regions and instability. Therefore, by tuning the cavity length and the flow field pressure, it is possible to effectively suppress the adverse effects and promote the positive effects of cavitation.

Cavitation number is an important parameter to describe the flow state of the cavitation process. In previous investigations^10–12^, the cavitation number is composed of the throat-part flow velocity *v*_*th*_, the density of water *ρ*_*l*_, the saturated vapour pressure of the water temperature *p*_*g*_, and the outlet pressure *p*_*out*_. In this paper, the corresponding flow conditions are described by the cavitation number *σ*, as expressed in Eq. (1):

1 $$\sigma { = }\frac{{p_{out} - p_{g} }}{{\frac{1}{2}\rho_{l} v_{th}^{2} }}$$

The length of an attached cavity reflects important information on the cavitation process, which has attracted substantial attention. Two types of studies, namely, external and internal cavitation studies, have been conducted in previous research on the cavity length. For external cavitation by hydrofoils, Kjeldsen^13^ experimentally investigated a 2D hydrofoil with an NACA 0015 cross-section and reported that the non-dimensional cavity length (*l*/*c*) increases linearly as the non-dimensional parameter 1/2 of the ratio of cavitation number to divergence angle (*σ*/2*α*) decreases. Mostafa^14^ analysed the effect of the cavitation number on the attached cavity length and thickness by numerical simulation of cavitation in a hydrofoil flow. They found that the cavity length and thickness increased synchronously with the decrease of the cavitation number. For internal cavitation by Venturis and Orifices, Sato^15^ observed the individual cavitation bubble length and the attached cavity length that correspond to various values of the cavitation number through a Micro-Venturi with a divergence angle (*θ* = 10°). Sayyaadi^16^ used the light intensity comparison technique to determine the attached cavity length in a Venturi flow and identified a typical trend, namely, the increase of the cavity length with the decrease of cavitation number. Long^17^ concluded from snapshots of the cavitating region in a Venturi channel that the dimensionless average cavity length is linearly related to the cavitation number and the pressure ratio. This relationship is also identified in the experimental study of Jahangsir^18^.

The fluctuation of the cavity length is mainly caused by the shedding of the cloud cavity. Two types of mechanisms, namely, re-entrant flow and pressure shock wave, lead to the shedding of the cloud cavity. In terms of external cavitation that is driven by a pressure wave, Leroux^19^ deemed that the pressure wave drives the cloud cavity to detach. Since the ratio of the cavity length to the hydrofoil chord length is greater than 0.5, the tail of the cavity violently fluctuates, which generates a strong pressure wave that propagates upstream and disconnects the attached cavity from the throat part. Regarding internal cavitation that is driven by re-entrant flow, Furness^20^ simulated the morphological change process of cloud cavity and found that the re-entrant flow occurs in the near-wall region, which is one of the important reasons for the instability of the cavity. By using a dual optical probe, Stutz^21^ verified that the re-entrant flow breaks up the attached cavity, thereby causing the shedding of the cloud cavity. Periodic fluctuations in the cavity length that are induced by the re-entrant flow were observed in the experimental study of Dular^22^. In addition, they qualitatively analysed the relationship between the Venturi throat part and the cavity length instability.

The morphological change of the cavity is often related to the pressure of the flow field. Many studies have been conducted on the pressure characteristics of cavitation, which mainly focus on the relationship between the pressure of the cavitation region and the dynamic behavior of cloud cavity. Considering external cavitation, Huang^23^ found that no substantial adverse pressure gradient exists in the near-wall cavitation region with a high value of the cavitation number. There is a substantial adverse pressure gradient in the near-wall cavitation region, and the cavity periodic shedding phenomenon at the tail of the hydrofoil causes periodic fluctuations of the hydrofoil lift coefficient with a low value of the cavitation number. According to the research of Ji^24^, the vorticity field that is caused by the mass transfer effect of the cavity is closely related to the pressure fluctuations on the hydrofoil surface based on numerical simulations. Considering the internal cavitation, Gopalan^25^ concluded from snapshots of the cavitating flow and pressure signals of the cavitating region in a Venturi that there is no re-entrant flow when the cavity thickness is very small. Moreover, the cloud cavity collapses directly in the downstream region. The relationship between the shedding of the cloud cavity and the pressure oscillation of the cavitation region was identified by Chen^26,27^. They indicated that the pressure fluctuations are gentle when the cavity is growing, and the pressure peak occurs when the shedding cavity collapses. From the simulation results of the cavitation process in the Venturi that were obtained by Charriere^28^, the propagation direction of the pressure wave that was generated by the shedding cavity collapse was identified, and they found that the propagation of pressure wave is closely related to the shedding cloud cavity. Based on the above, the research related to Venturi cavitation was summarized in Table 1^15,17,18,20,21,25–28^.

**Table 1:** Previous experimental study on Venturi cavitation^15,17,18,20,21,25–28^.

References	Cavitation number ***σ***	Reference speed *v*_*ref*_ /m·s^−1^	Reference pressure *p*_*ref*_ /kPa	Divergence angle ***θ***/°	Research content
Sato^15^	0.5–1.2	12.74–21.44(*v*_*th*_)	43–278(*p*_*th*_)	5°	cavity snapshot; cavitation process noise
Long^17^	0.2–1.0	15.02–27.12(*v*_*th*_)	91–378(*p*_*out*_)	6°	average cavity length
Jahangir^18^	0.3–1.0	7.45–26.82(*v*_*th*_)	30–110(*p*_*out*_)	8°	cavity length and structure
Furness^20^	1.2–2.5	*v*_*th*_	*p*_*in*_	8°	cavity length; pressure oscillation
Stutz^21^	1.997	10.36–15.47(*v*_*th*_)	109–249(*p*_*in*_)	8°	numerical simulation; cavity image; re-entrant flow
Gopalan^25^	4.4, 4.7	3–10(*v*_*th*_)	22–237(*p*_*in*_)	12°	void fraction and velocity distribution in cavitation area
Chen^26^	1.2, 1.8	10.4(*v*_*th*_)	67–100(*p*_*out*_)	10°	cavity thickness; transient velocity field
Chen^27^	0.7–1.1	10.52(*v*_*th*_)	40–63(*p*_*out*_)	10°	cavity length; pressure oscillation
Charriere^28^	2.1–2.2	7.04(*v*_*th*_)	55(*p*_*in*_)	8°	cavity shedding; cavity instability

where *p*_*th*_ is the pressure of the Venturi throat part and *p*_*in*_ is the pressure of the Venturi inlet.

Previous works indicated that structure and dimensions of a Venturi cavitator influenced cavity length^29–31^, breakup^22^ and pressure oscillation^32,33^. Divergence angle significantly changes the flow condition in a Venturi channel, thus affects the performance of a Venturi cavitator, while little research has been reported about the divergence angle of the Venturi or the pressure of the entire flow field in the divergent part. Therefore, in this study, the behavior of the liquid/vapor interface in Venturis with various divergence angles was investigated by using a high-speed camera, and the wall static pressure of entire flow field of the divergent part was synchronously measured. Then, images of various experimental conditions were captured and used to analyse the influences of the divergence angle on the cavity length. The coupled relationships between the liquid/vapour interface and the dynamic pressure were used to identify the pressure oscillation mechanism of cavitation in Venturis with various divergence angles.

## Results

### Typical cloud cavity shedding patterns

According to the experimental snapshots, the sizes of the attached cavity and the shedding cloud cavity differ substantially under all experimental conditions. Three typical cavitation shedding patterns are identified based on the following criteria: (I) front shedding (0 ≤ *x*_*s*_/*L*_*max*_ ≤ 0.3); (II) central shedding (0.3 ≤ *x*_*s*_/*L*_*max*_ ≤0.7); and (III) tail shedding (0.7 ≤ *x*_*s*_/*L*_*max*_ ≤1.0). *x*_*s*_/*L*_*max*_ is the ratio of the initial shedding position of the cloud cavity (*x*_*s*_) to the maximal length of the attached cavity prior to shedding (*L*_*max*_).

#### (1) Front shedding

**Figure 1:**
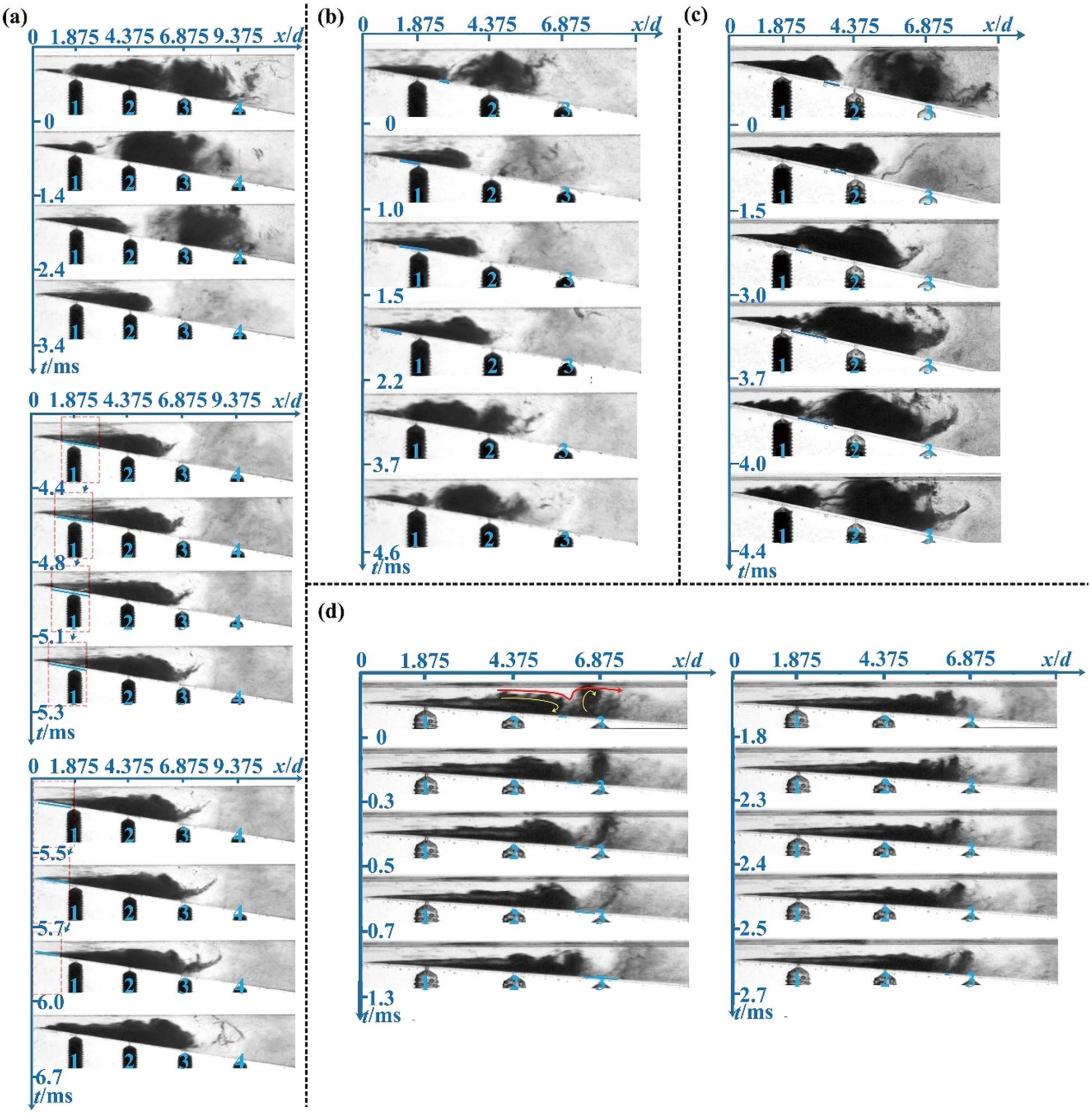
(**a**) (*v*_*th*_ = 18 m·s^-1^, *σ* = 0.45, and *θ* = 10°) Front shedding of the cavity (Type I). (**b**) (*v*_*th*_ = 18.8 m·s^-1^, *σ* = 0.65, and *θ* = 10°) Central shedding of the cavity (Type II). (**c**) (*v*_*th*_ = 19.6 m·s^-1^, *σ* = 0.65, and *θ* = 12°) Central shedding of the cavity (Type II). (**d**) (*v*_*th*_ = 21.9 m·s^-1^, *σ* = 0.55, and *θ* = 4°) Tail shedding of the cavity (Type III). In the above images, *x* is the distance to the Venturi throat part, *d* is the diameter of the Venturi throat part and *t* is the image corresponding moment.

As shown in Fig. 1a, the typical front shedding process can be divided into three stages: (1) In the first stage (*t* = 0–3.4 ms), the attached cavity extends downstream and the shedding cloud cavity gradually collapses. (2) In the second stage (*t* = 3.4–5.7 ms), a low vapour fraction region inside the red dotted box begins to appear in the attached cavity and, thereafter, moves upstream to the throat part, which is represented by the near-wall blue line in Fig. 1a. (3) In the third stage (*t* = 5.7–6.7 ms), the attached cavity splits into a large-scale shedding cloud cavity and an initial small-scale attached cavity at the throat part. The remarkable feature of front shedding progress is that the distance *x*_*s*_ from the throat part to the cloud cavity initial shedding point is less than 30% of the maximum length *L*_*max*_ before the attached cavity detaches. The front shedding is periodic and initially occurs in the throat part or near the throat part. Ganesh^34^ have also captured the phenomenon of front shedding via high-speed visualization technology and time-resolved X-ray densitometry measurements.

#### (2) Central shedding

The central shedding (II) is driven by two different shedding mechanisms. One is based on the pressure wave mechanism. As shown in Fig. 1b, the typical central shedding process is similar to the front shedding process. The only difference is the third stage (*t* = 2.2–4.6 ms), in which the attached cavity doesn’t break at the throat part, but continues to grow. The region of the attached cavity that was previously connected to throat part becomes increasingly thin and finally vanishes in the downstream high-pressure region. The entire cavity detaches from the middle as observed by Stutz^35^. The other is based on the re-entrant flow mechanism. As shown in Fig. 1c, the shedding process can still be divided into three stages: (1) In the first stage (*t* = 0–1.5 ms), the attached cavity gradually grows, and the shedding cavity begins to collapse. The re-entrant flow appears at the lower wall surface, which corresponds to the near-wall blue line in Fig. 1c. (2) In the second stage (*t* = 1.5–3.0 ms), the re-entrant flow moves upstream along the lower wall to the middle of the attached cavity. (3) In the third stage (*t* = 3.0–4.4 ms), this re-entrant flow breaks up the shedding cloud cavity from the middle of the attached cavity. This shedding progress is driven by the water jet formed by the reverse pressure gradient. However, the re-entrant flow moves upstream only for a short distance, which produces a middle-scale shedding cavity. The remarkable feature of central shedding pattern is that the distance *x*_*s*_ from the throat part to the cloud cavity initial shedding point is between 30–70% of the maximum length *L*_*max*_ before the attached cavity detaches. The central shedding is periodic and initially occurs in the centre of the cavity.

#### (3) Tail shedding

Figure 1d shows a typical tail shedding process (*t* = 0–2.7 ms). The attached cavity grows downstream from the throat. Due to the small divergence angle or high cavitation number, an inverted conical cavity with a thicker tail and a thinner front is formed. The tail of the cavity occupies most of the cross-section of the divergent part. Tail shedding appears under the shock of the water flow represented by the red line in Fig. 1d. The principles of driving the cavity tail shedding are as follows: First, the water circulation cross-section changes from wide to narrow, part of the momentum of the flowing water is transferred to the tail part of the cavity. The tail part of the cavity drifts faster in the flow direction than the front part and the tail shedding occurs under the effect of the speed difference. Secondly, at the wide water flow cross-section in the middle, the liquid-phase water above the gas–liquid interface undergoes a counter-clockwise swirl. Combining relative motion and viscous effects, the front part of the cavity and the tail part of the cavity at here have opposite rotations (shown by the yellow arrows in Fig. 1d), which exacerbates the fracture at the intermediate junction and in turn causes the tail shedding process. The most prominent feature of tail shedding is that the distance *x*_*s*_ from the throat part to the cloud cavity initial shedding point is greater than 70% of the maximum length *L*_*max*_ before the attached cavity detaches. The tail shedding initially occurs in the tail of the cavity and exhibits no readily observable periodicity. Pawar et al.^22,36^ used high-speed photography to observe a similar cavity tail shedding process.

**Figure 2:**
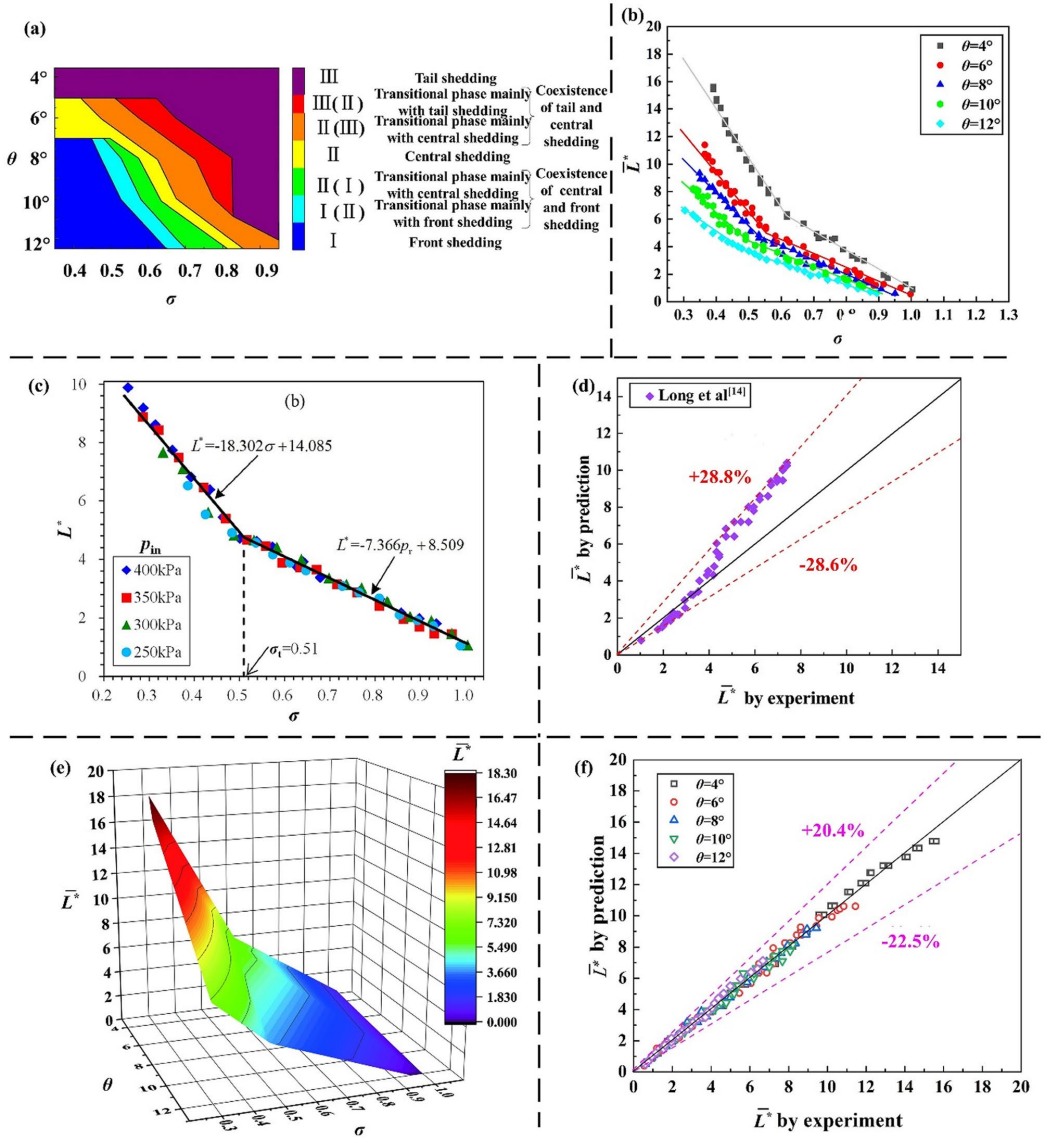
(**a**) Shedding pattern regimes under various experimental conditions. (**b**) (*σ* = 0.3–1.0 and *θ* = 4–12°) The dimensionless average cavity length ${\stackrel{-}{L}}^{*}$ versus the cavitation number and the divergence angle. (**c**) (*θ* = 6°) The dimensionless cavity length ${\stackrel{-}{L}}^{*}$ versus the flow parameters by Long^17^. (**d**) (*θ* = 6°) The dimensionless average cavity length ${\stackrel{-}{L}}^{*}$ that is predicted by the correlation of Long^17^. (**e**) Average cavity length ${\stackrel{-}{L}}^{*}$ by the Venturi with different divergence angles in the cavitation number (*σ = *0.3–1.0). (**f**) Comparison of the experimental and predicted values of the average cavity length ${\stackrel{-}{L}}^{*}$.

The shedding behavior of the attached cavity has a strong influence on the morphological changes of cavity. Through the analysis and statistics of the cavity shedding types under various experimental conditions, the relationship of the shedding type with the divergence angle and the cavitation number is identified. Comparing Table 2 with Fig. 2a, as the divergence angle increases from 4° to 12°, the cavity shedding type transitions from III to II to I. The initial shedding position gradually moves from the tail of the attached cavity to the front, and the scale of the shedding cavity increases rapidly. For the divergence angle is 4°, tail shedding (III) mainly occurs in a cavitation process with various flow parameters. For the divergence angle is 6°, the shedding type changes from III to II as cavitation number decreases. For the divergence angle is 8°, 10°, or 12°, the shedding type changes from III to II to I as cavitation number decreases. And with the decrease of the cavitation number, there is an intermediate transition process between two different shedding types. This is mainly a state in which two shedding types coexist. For example, the intermediate transition process in the transition from III to II includes: first, the transitional phase mainly with tail shedding (III/II), and second, the transitional phase mainly with central shedding (II/III). For the cavitation characteristics in the Venturis with the shedding type transition (divergence angles of 6°, 8°, 10°, and 12°), as the divergence angle increases, the range of cavitation number corresponding to the above-mentioned intermediate transition process gradually moves to the high cavitation number region.

**Table 2:** (*σ* = 0.40–0.90 and *θ* = 4–12°) Statistics on the cavity shedding types.

*σ*	4°	6°	8°	10°	12°
0.90	III	III	III	III	II/III
0.85	III	III	III	III	II/III
0.80	III	III	III/II	III/II	II
0.75	III	III	III/II	II/III	II/I
0.70	III	III	II/III	II/III	II/I
0.65	III	III/II	II/ III	II	I/II
0.60	III	III/II	II	II/I	I
0.55	III	II/III	II/I	I/II	I
0.50	III	II/III	I/II	I	I
0.45	III	II	I	I	I
0.40	III	II	I	I	I

### Length of the cavity

#### (1) Cavity length analysis

According to a Venturi cavitation device, the average length of cavity is the most important parameter in applications. Prediction of averaged cavity length is a key issue in both theoretical and experimental research. As shown in Fig. 2b, for different divergence angles (*θ* = 4°, 6°, 8°, 10° and 12°), the average cavity length increases slowly initially and subsequently rapidly with the decrease of cavitation number. Among all divergence angles, there is an inflection point of the growth rate of the average cavity length. Under the corresponding experimental conditions of the inflection point, the upper part of the cavity begins to contact the upper wall surface of the divergent part. For various values of the cavitation number, the average cavity length decreases with the increase of the divergence angle and the inflection point of the growth rate of the average cavity length gradually moves to the low cavitation number region as the divergence angle increases.

#### (2) Cavity length prediction

Long^17^ introduced the relationship between the dimensionless parameters, the cavitation number and the cavity average length for a Venturi with a divergence angle (*θ* = 6°). As plotted in Fig. 2c, the relationship is expressed as follows:

2 $$\left\{ \begin{gathered} \overline{L}^{*} = - 7.366\sigma + 8.509{ 0}{\text{.51}} \le \sigma \le 1.0 \hfill \\ \overline{L}^{*} = - 18.302\sigma + 14.085{ 0}{\text{.2}} \le \sigma \le 0.51 \hfill \\ \end{gathered} \right.$$

As shown in Fig. 2d, the correlation by Long^17^ is used to predict the experimental data. The prediction errors are in the range of − 28.6% to + 28.8% for all test conditions (*θ* = 6°). Since the average cavity length is not only affected by the cavitation number but also is related to the divergence angle. Therefore, combined with the effect of divergence angle, the Eq. 3 is transformed to:

3 $$\left\{ \begin{gathered} \overline{L}^{*} = { - 27}{\text{.052}}\theta^{{{ - 0}{\text{.489}}}} \sigma + {33}{\text{.471}}\theta^{{{ - 0}{\text{.592}}}} { (}\sigma_{i} \le \sigma \le {1}{\text{.0,4}} \le \theta \le {12)} \hfill \\ \overline{L}^{*} = { - 102}{\text{.636}}\theta^{{{ - 0}{\text{.692}}}} \sigma + {88}{\text{.978}}\theta^{{{ - 0}{\text{.783}}}} { (0}{\text{.3}} \le \sigma \le \sigma_{i} {,4} \le \theta \le {12)} \hfill \\ \sigma_{i} = \frac{{{88}{\text{.978}}\theta^{{{ - 0}{\text{.783}}}} - {33}{\text{.471}}\theta^{{{ - 0}{\text{.592}}}} }}{{{102}{\text{.636}}\theta^{{{ - 0}{\text{.692}}}} - {27}{\text{.052}}\theta^{{{ - 0}{\text{.489}}}} }}{ (4} \le \theta \le {12)} \hfill \\ \end{gathered} \right.$$

where *σ*_*i*_ is the cavitation number corresponding to the inflection points.

With consideration of the effects of the divergence angle and the cavitation number, a three-dimensional relation are shown in Fig. 2e. As mentioned above, both the cavitation number and the divergence angle play a significant role in Venturi cavitation. According to the effect of cavitation number on cavity length, whether at low cavitation number or at high cavitation number, the cavity average length ${\stackrel{-}{L}}^{*}$ increases approximately linearly with the decrease of the cavitation number. And there are inflection points in the growth rate for different divergence angles in the middle cavitation number region. For the effect of the divergent angle on cavity length, at low cavitation number, the average cavity length increases with the decrease of the divergence angle, and the divergence angle contributes a larger impact in cavity growth process in the case of low cavitation number.

Comparing the experimental and predicted values of the average cavity length, as plotted in Fig. 2f, the new correlation more accurately predicts the length of the cavity. The predicted errors are in the range of -22.5% to + 20.4%.

### Relationship between the transient fluctuation characteristics of the wall static pressure and the morphological changes of cavitation

#### (1) Front shedding/central shedding

**Figure 3:**
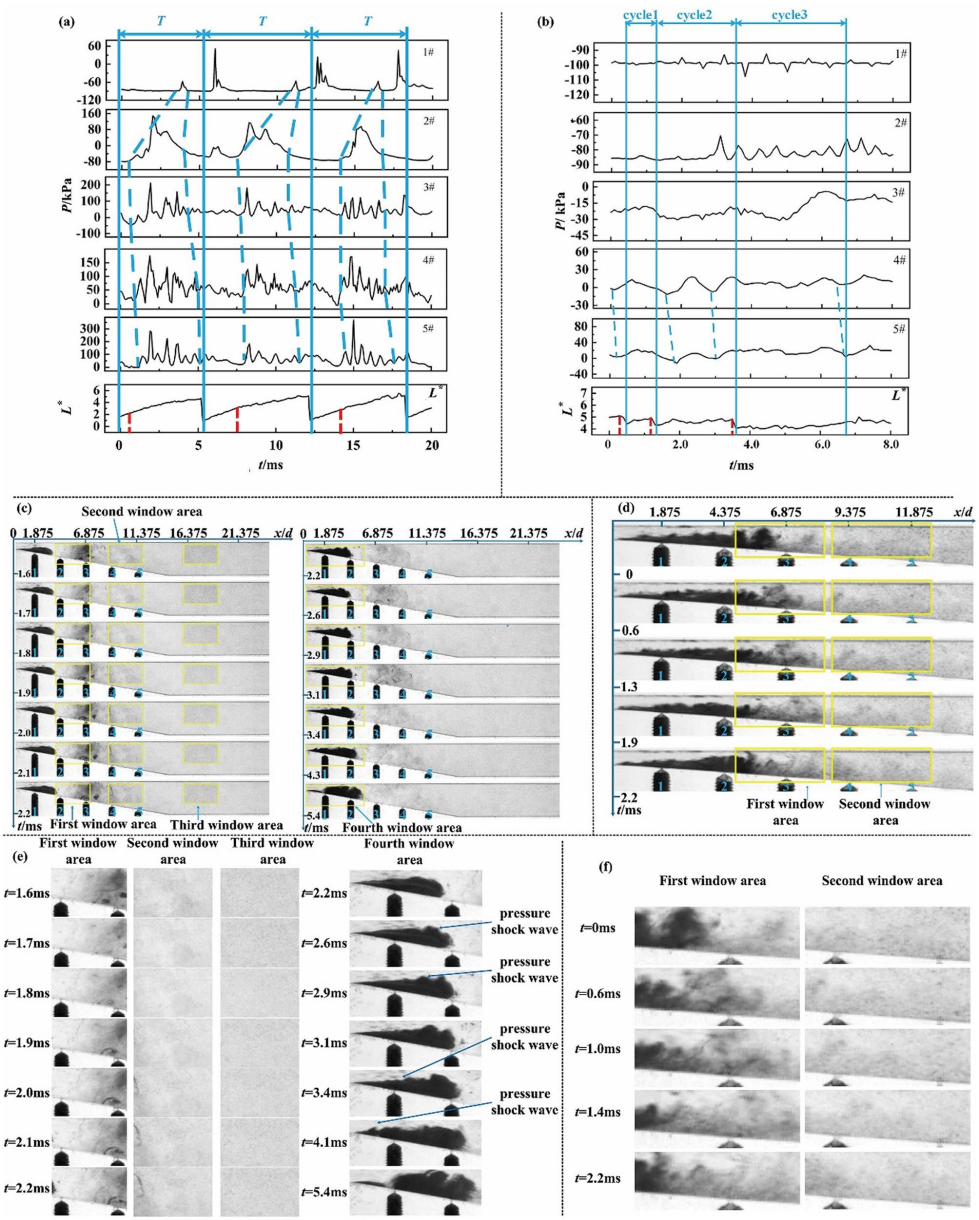
(**a**) (*σ* = 0.55 and *θ* = 10°) Pressure fluctuations and cavity length fluctuations during the front shedding cycle *T* (three cycles). (**b**) (*σ* = 0.6 and *θ* = 4°) The pressure fluctuations and cavity length fluctuations in tail shedding (I) (three cycles). (**c**) (*σ* = 0.55 and *θ* = 10°) Viewport in the images that correspond to the first cycle. (**d**) (*σ* = 0.6 and *θ* = 4°) Viewport in the images that correspond to the second cycle. (**e**) Enlarged image at each transducers of the first cycle. (**f**) Enlarged image at each transducers of the second cycle.

The wall static pressure fluctuations and cavitation images of the divergent part are obtained by the synchronous triggering system. Combined with the wall static pressure fluctuation characteristics of each transducer and the transient cavitation behaviour, the process of front shedding (I) is shown in Fig. 3a,c,e. The blue dashed lines in Fig. 3a describe the direction of pressure wave propagation and the red dashed lines represent the time at which the cavity begins to collapse. The blue solid lines divide the wall static pressure fluctuations into three periods. According to the first period (0–5.8 ms), the pressure wave peak caused by the collapse of the cloud cavity generates near the transducer #3 at *t* = 1.7 ms, and it propagates in both upstream and downstream directions. The pressure peak time and fluctuation amplitude corresponding to each transducer are divided into two categories: (1) Transducer #2 (*t* = 1.8 ms) located upstream and transducer #4, #5 (*t* = 1.7 ms) located downstream. The pressure fluctuation amplitude Δ*P* is about 240 kPa, which is basically the same as the pressure peak time and fluctuation amplitude Δ*P* at the collapse of the cavity. It indicates that the pressure wave propagation speed is fast and there is almost no attenuation during the propagation process. (2) Transducer #1 (*t* = 4.0 ms) located upstream. The pressure peak time is far behind the time of the pressure peak appearing. The pressure fluctuation amplitude Δ*P* is about 30 kPa, which is greatly reduced. It indicates that the pressure wave propagation speed is small and the attenuation during the propagation process is obvious. As shown in the yellow window in Fig. 3c, the first window (transducers #2, #3), the second window (transducers #4, #5), the third window (downstream of the divergence section), and the fourth window (transducer #1) have been selected. The cavitation images at each transducers are analyzed with time series and the corresponding enlarged view is shown in Fig. 3e. At *t* = 1.6–1.7 ms, the grayscale of the first window (transducers #2, #3) and the second window (transducers #4, #5) suddenly decreases, which is consistent with the moment of pressure peaks at the transducers 2, 3, 4, and 5. This indicates that the local steam condenses into a liquid state during the pressure wave propagation process, which in turn results in a sudden decrease in the gas content in the area. At *t* = 1.7–1.8 ms, the grayscale of the third window area suddenly decreases, indicating that the pressure wave propagates downstream of the divergence section with a large propagation speed. At *t* = 3.4–4.1 ms, the upper gas–liquid interface of the attached cavity at the transducer #1 in the fourth area protrudes, as shown by the yellow arrow, which is consistent with the location of pressure wave peak (transducer #1). Comparing Fig. 3a,e, it can be found that the cavitation image is consistent with the pressure fluctuation. The propagation speed and fluctuation amplitude of the pressure wave in the liquid phase region (transducers #2–5) are much higher than those in the gas phase region (transducer #1). It is related to the attenuation of pressure wave in the gas phase region. At the same time, the propagation of the pressure wave in the liquid phase region is reflected as a decrease in the local gas content in the cavitation images, and the propagation of the pressure wave in the gas phase region is reflected as the deformation of the cavity interface in the cavitation images. The pressure wave speed calculated from the time each transducer reaches the peak of pressure is about 600 m/s, which is close to the value calculated by Jahangir from the image processing^18^. The pressure of the divergent part in the process of central shedding (II) exhibits a periodic large-scale oscillation that is similar to that of the front shedding (I).

#### (3) Tail shedding

As shown in Fig. 3b,d,f, in the tail shedding (III) process (0–1.3 ms), the wall static pressure and the cavity length fluctuate over time with no periodicity. It can be found that the cloud cavity collapsed at the transducer #3 at *t* = 0.6 ms, and the pressure wave propagates upstream and downstream. The moments of the pressure wave peak and fluctuation ranges corresponding to each transducers are divided into two types: (1) Transducers #1, #2 located upstream. No obvious pressure wave is observed because the pressure wave generated by the small-scale shedding cavity is reduced, and it is attenuated and annihilated in the attached cavity during the upstream propagation. (2) Transducer #4 (*t* = 0.7 ms) and transducer #5 (*t* = 0.8 ms) located downstream. The pressure fluctuation amplitude ΔP is about 15 kPa, which is smaller than the front shedding. The time of the pressure wave peak at transducer #4, #5 behind the time of pressure wave peak appearing. It indicates that the pressure wave propagation speed is small and the attenuation during the propagation process in high gas content area also exists. As shown in Fig. 3f. At *t* = 0–0.6 ms, the grayscale (gas content) of the first window (transducer #3) suddenly decreases, which is consistent with the moment when the pressure peak appears at the transducer #3. At *t* = 0.6–1.0 ms, the grayscale of the second window area suddenly decreases. Comparing the three types of shedding processes that are discussed above and the wall static pressure fluctuation characteristics, for a larger divergence angle (*θ* = 10°), the shedding types of cavity are concentrated in front shedding and central shedding, which are accompanied by large-scale shedding cavity. The shedding cavity collapses to generate high-intensity pressure wave. The intensity of the pressure wave remains high throughout the downstream liquid-phase region. The pressure wave propagates through the cavity with a high vapour fraction (Fig. 1a) or local boundary deformation (Fig. 3e), and the intensity is rapidly attenuated. However, the shedding types of cavity are concentrated in tail shedding in the Venturi with a smaller divergence angle (*θ* = 4°), and the small-scale shedding cloud cavity produced by tail shedding collapses to generate a pressure wave with little intensity.

### The wall static pressure fluctuation characteristics over time

#### (1) The wall static pressure spectrum analysis

**Figure 4:**
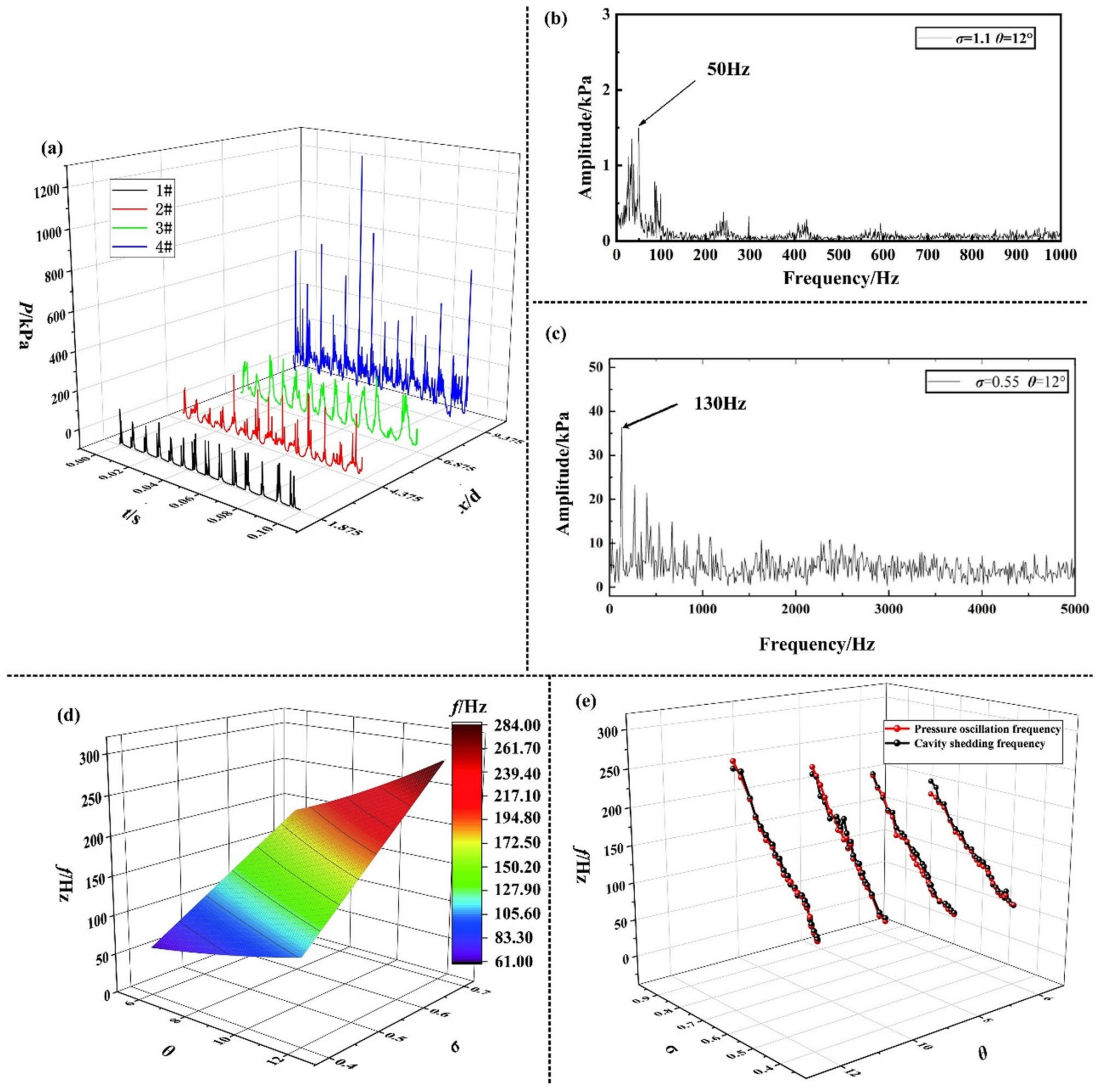
(**a**) (*σ* = 0.45 and *θ* = 12°) The wall static pressure fluctuation versus time. (**b**) (*σ* = 1.1 and *θ* = 12°) The wall static pressure oscillation frequency *f* obtained through FFT processing. (**c**) (*σ* = 0.45 and *θ* = 12°) The wall static pressure oscillation frequency *f* obtained through FFT processing. (**d**) Relation of the wall static pressure oscillation frequency *f* with the cavitation number *σ* and the divergence angle. (**e**) Comparison of the wall static pressure oscillation frequency and the cavity shedding frequency.

According to Fig. 4a, for the cavitation process in the Venturi with a divergence angle (*θ* = 12°), the wall static pressure fluctuation exhibits very strong periodicity in each transducers and the pressures at each position of the divergent part have the same oscillation frequency. The natural oscillation frequency of the system is the oscillation frequency of the entire test system under non-cavitation experimental condition, which is 50 Hz in this experimental system as shown in Fig. 4b. The experimentally obtained wall static pressure signals are processed via the Fast Fourier Transform, as shown in Fig. 4c. It is found that the wall static pressure oscillation frequency (130 Hz) is very similar to the cavity shedding frequency (129 Hz).

#### (2) The wall static pressure oscillation frequency

Based on the comprehensive analysis of the cavity length fluctuation characteristic and the wall static pressure spectrum of each transducer under various conditions, it can be obviously found that the cavity shedding frequency and the wall static pressure oscillation frequency are very consistent in Venturis with divergence angle (*θ* = 6°, 8°, 10° and 12°) from Fig. 4e, but there is no obvious pressure fluctuation frequency in the Venturi with small divergence angle (*θ* = 4°). For the larger cavitation number (0.9 > *σ* > 0.7), The tail shedding still exists. After filtering the pressure signal, it is found that there also is no obvious fluctuation frequency. For the smaller cavitation number (0.7 > *σ* > 0.3), the pressure oscillation frequency shows a clear regularity. To analyse the correlations of the wall static pressure oscillation characteristics with the cavitation number (0.7 > *σ* > 0.3) and the divergence angles (*θ* = 6°, 8°, 10° and 12°), the oscillation frequency *f* of the wall static pressure signals in the Venturis are analysed, as shown in Fig. 4d. For various divergence angles, the wall static pressure oscillation frequency *f* follows the same variation law with the cavitation number, namely, the oscillation frequency increases in an approximately linear relationship with the increase of the cavitation number. This is mainly due to the transition of the cavity shedding type from III to II to I, and the increase in the scale of the shedding cloud cavity and the amplitude of the collapsing pressure. For various cavitation numbers, the variation laws of the wall static pressure oscillation frequency are similar, namely, the oscillation frequency increases with the increase of the divergence angle, which can also be explained by the increase in the scale of the shedding cloud cavity and the amplitude of the collapsing pressure as the divergence angle increases.

## Discussion

In this study, an experiment was conducted to investigate the cavity length and the wall static pressure characteristics at various flow parameters through rectangular convergence-divergence Venturis with divergence angles of 4°, 6°, 8°, 10° and 12°. Several conclusions were drawn:

1. The cavitation characteristics of Venturis with various divergence angles were investigated at cavitation numbers that ranged from 0.3 to 1.0 based on the synchronous analysis of the vapour–liquid interface and its corresponding wall static pressure. Three types of cavity shedding regimes, namely, front shedding (I), central shedding (II), and tail shedding (III), were defined in the experimental conditions. Front shedding (I) and central shedding (II) exhibited strong periodicity. Moreover, a shedding type map of the cavitation number and the divergence angle was plotted.
2. In regard to different divergence angles (*θ* = 4°, 6°, 8°, 10° and 12°), the cavity length increased slowly initially and subsequently rapidly increased with the decrease of the cavitation number. A dimensionless correlation between the cavitation number and the divergence angle was identified and used to predict the average cavity length. The prediction errors were in the range of − 22.5% to + 20.4%.
3. For a larger divergence angle (*θ* = 10°), the shedding types of cavity were concentrated in front shedding and central shedding, which were accompanied by high-intensity pressure waves. For a smaller divergence angle (*θ* = 4°), the shedding types of cavity were concentrated in tail shedding, accompanied by little-intensity pressure waves.
4. The wall static pressure oscillation frequency of cavitation in the Venturis with large divergence angles (*θ* = 6°, 8°, 10° and 12°) was approximately equal to the cavity shedding frequency. The oscillation frequency increased with the increase of the cavitation number and the divergence angle. However, the wall static pressure fluctuation of cavitation in the Venturi with small divergence angle (*θ* = 4°) exhibited no readily observable periodicity.

## Methods

### Experimental apparatus

**Figure 5:**
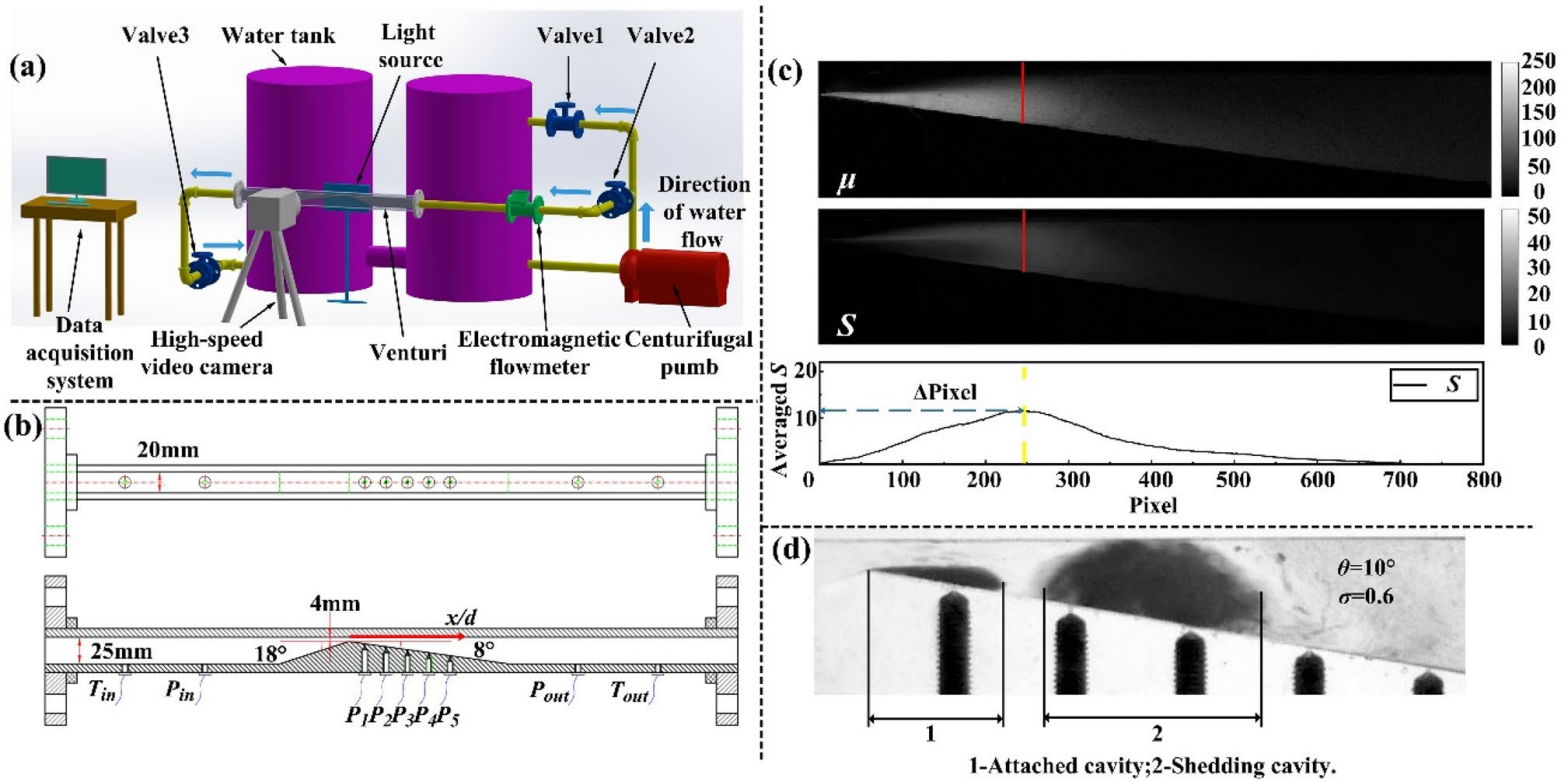
(**a**) Schematic diagram of the experimental system of the Venturi cavitation. (**b**) Structural parameters of the convergence–divergence Venturi with a rectangular cross-section. (**c**) Correspondence between the average cavity length position, the maximum average standard deviation, and the peak of the standard deviation curve. (**d**) Two types of cavities.

As illustrated in Fig. 5a, the experimental system consisted of several main components, such as a water tank, a centrifugal water pump, water pipes, a electromagnetic flowmeter, control valves, Venturi test sections, data acquisition systems, a high-speed video camera and a light source. The water flow line in the experiment is described as follows: First, water was delivered by the centrifugal water pump from the water tank to the rear waterway at a constant flow rate. Then, a portion of the water flowed back from the three-way section to the water tank, and another portion of the water flowed from the three passes to the Venturi experimental section. Eventually, this portion of the water returned to the water tank after cavitation in the Venturi divergent part. The experimental method for maintaining pressure and flow rate is elaborated as follows: Referring to Fig. 5a, first keep valve 2 and valve 3 fully open, and adjust valve 1 to a certain opening, so that the flow rate is stable at 5 m^3^·h^−1^ and the downstream pressure *p*_*out*_ is also kept constant. According to formula (1), the cavitation number ***σ*** is also constant. Then, the opening of valve 1 is continue reduced. The water flow rate through the Venturi experimental section of the test circuit is continuously changed from 5 m^3^·h^−1^ to 9 m^3^·h^−1^ (the venturi throat flow rate is continuously increasing) and the downstream pressure of the experimental section is also increasing. Combination with the power exponents of the venturi throat flow rate and downstream pressure when calculating the cavitation number in formula (1), as the flow rate increases, the cavitation number ***σ ***decreases.

Figure 5b specifies the geometric parameters and illustrates the transducer placement of the rectangular Venturi with a divergence angle (*θ* = 8°). The test sections were five types of Venturis with various divergence angles, which were made of organic glass and exhibited satisfactory light transmittance. The flow direction was from left to right, and the rectangular cross-section was 20 mm × 25 mm. Since the cross-section of the Venturi is rectangular, the three-dimensional Venturi cavitation was equivalent to two-dimensional cavitation. The vertical spacing of the throat part was equivalent to the Venturi throat radius. The throat part radius *r* was 4 mm and the length of the throat part was 0 mm. The convergence angle was 18° and the divergence angles were 4°, 6°, 8°, 10°, and 12°, respectively. As illustrated in Fig. 5b, the positions in the test sections are characterized by values x^*^ = *x*/*d*, non-dimensionallized using the diameter of the Venturi throat which equalled twice the throat radius *r*. The outlet and inlet temperature transducers were installed at *x*/*d* = 38.75 and -26.25, respectively. The outlet and inlet pressure transducers were installed at *x*/*d* = 29.375 and -16.875, respectively. The dynamic pressure transducers in divergent part, which were labelled #1–6, were installed at *x*/*d* = 1.875, with the step size of 2.5. The parameters of the Venturi experimental section are listed in Table 3.

**Table 3:** Structural parameters of the test sections.

Parameter	Section 1	Section 2	Section 3	Section 4	Section 5
Venturi length/mm	780	780	650	650	650
Divergence angle *θ*/°	4	6	8	10	12
Radius of the throat part *r*/mm	4	4	4	4	4
Number of pressure transducers in the divergent part	5	5	5	6	6

### Experimental methods

The water volume flux was controlled by three control valves and measured by an electromagnetic flowmeter (with a range of 0–12 m^3^·h^−1^ and an accuracy of 0.5%FS). The static pressure signals at the inlet and outlet of the Venturi were measured by using a static pressure transducer (with a range of 0–1.0 MPa and an accuracy of 0.25%FS). Two K-type thermocouples (with a range of 0–100 °C and an accuracy of 0.5%FS) were installed on the inlet and outlet of the Venturi for measuring the water temperature, which respectively corresponded to *T*_*in*_ and *T*_*out*_ in Fig. 5b. The experimental temperature was defined as the average value of the Venturi inlet and outlet temperatures. The pressure fluctuation signals that were caused by cavitation were measured by dynamic pressure transducers#1–#2 (gauge pressure with a range of − 100–1,900 kPa, an accuracy of 0.5%FS and a response frequency of 10,000 Hz) and dynamic pressure transducers#3-#6 (gauge pressure with a range of − 100–3,900 kPa, an accuracy of 0.5%FS and a response frequency of 10,000 Hz). And their values respectively corresponded to the value of *P*_*1*_ to *P*_*6*_. The high-speed camera that was used in the cavitation experiments was Phantom v611 with 0.12 megapixels. The synchronous triggering system was used to simultaneously record the wall static pressure signals and imaging the cavitation zone. The recording time was set to at least 10 s, and the sampling rate was 10,000 Hz. The detailed test conditions are listed in Table 4.

**Table 4:** Test conditions of the Venturi cavitation.

Parameter	Value	Uncertainty
Water temperature/°C	16.6–18.2	0.030
Inlet pressure, *p*_*in*_/kPa	167.20–372.26	0.015
Outlet pressure, *p*_*out*_/kPa	129.08–186.86	0.019
Water volume flux, *Q*/ m^3^·h^−1^	5.00–9.00	0.012
Reynolds number *Re*	1.092 × 10^5^–1.965 × 10^5^	0.012
Cavitation number *σ*	0.30–1.00	0.026
Width of the cross-section/mm	19.98–20.08	–
Radius of the throat part, *r*/mm	3.98–4.01	–
Sample frequency, Frame speed of the camera/Hz	10,000	–
Cylindrical water tank, diameter of the bottom face × height, /mm	1,000 × 2,500	–
Ambient pressure/kPa	101.325	–

### Experimental image post-processing

Determining the length of the attached cavity from the experimental images requires a series of image processing techniques. First, as illustrated in Eq. 4, the rgb2gray function in MATLAB software was used to convert the 8-bit colour original image *I* into a greyscale image, and the corresponding greyscale matrix was concluded by elements with 256 grey level (0 for black pixel and 255 for white pixel). Second, based on the Eq. 5, using a reference non-cavitation greyscale image *I*_*ref*_ to normalize the image *I*. The normalization was conducted by the calculation of (*I*_*ref*_-*I*)/*I*_*ref*_. Third, a trapezoidal structure was used to extract the image of the Venturi divergent part and the grey levels of elements outside the trapezoidal flow region in the image was changed to zero to save the calculations. Fourth, calculated the average value of each element in the grey matrix transformed from picture 1 to picture *I* as Eq. 6^37^. According to the obtained average grayscale matrix, combined with Eq. 7, a grayscale standard deviation matrix from picture 1 to picture *I* was obtained^38^. Fifth, verified the independence of the number of images required to obtain the average cavity length with respect to *I* = 50, 150, 250, 350, 450 and 550. When the grayscale average value and standard deviation of the images begin to stabilize with the increase of the number *I* of the processing images, it is determined that the number *I* used at this time is the most suitable number of image processing with high accuracy and small amount of calculation^17^. The optimal image processing number of the experimentally obtained images is *I* = 450. The corresponding uncertainty of the average value is less than 0.5% and the corresponding uncertainty of the gray standard deviation is less than 2.1%. Finally, as shown in Fig. 5c, the transitions between cavitation and non-cavitation were most frequent at the average cavity length position, so its standard deviation value is the greatest. Thus obtain the average value of the elements in each column of the grayscale standard deviation matrix to determine the position of the maximum value of the grayscale standard deviation, and calculate the cavity length by combining the corresponding scale.

4 $$X\left( {m,n,I} \right) \in \left[ {0,1 \cdots 255} \right]$$

5 $$X(m,n,I)=\left[ X_{ref} (m,n,I)-{X(m,n,I) } \right]/X_{ref} (m,n,I) = \left\{ {\left[ \begin{array}{*{20}c} x_{ref} (1,1,I) & \cdots & x_{ref} (1,n,I)  \\ \vdots  & \ddots & \vdots \\ x_{ref} (m,1,I) & \cdots &x_{ref} (m,n,I) \end{array} \right]-\left[ \begin{array}{*{20}c} x(1,1,I) & \cdots & x(1,n,I) \\ \vdots & \ddots & \vdots  \\ x(m,1,I) & \cdots & x(m,n,I) \end{array} \right]} \right\}/\left[ \begin{array}{*{20}c} x_{ref} (1,1,I)& \cdots& x_{ref} (1,n,I)  \\ \vdots  & \ddots &  \vdots \\  x_{ref} (m,1,I) & \cdots& x_{ref} (m,n,I)  \end{array} \right]$$

6 $$\mu (m,n) = \frac{1}{I}\sum\limits_{i = 1}^{I} {X(m,n,I)}$$

7 $$S(m,n) = \sqrt {\frac{1}{I - 1}\sum\limits_{i = 1}^{I} {\left[ {X(m,n,I) - \mu (m,n)} \right]^{2} } }$$

where *I* corresponds to the number of images to calculate the average cavity length, *X*(*m*,*n*,*I*) corresponds to the grayscale matrix of the image *I*, *x*(*m*,*n*,*I*) corresponds to the elements in m rows and n columns of the grayscale matrix of the image *I*, *μ*(*m*,*n*) corresponds to the average grayscale matrix from image 1 to image *I* and *S*(*m*,*n*) corresponds to the grayscale standard deviation matrix from image 1 to image *I*.

In the cavitation grayscale images, an attached cavity that is connected to the throat part and a separated shedding cavity in the downstream region of the divergent part are identified as shown in Fig. 5d.

## References

[1] Slater TJ (2017). Multiscale correlative tomography: an investigation of creep cavitation in 316 stainless steel. Sci. Rep..

[2] Garcia R, Hammitt FG (1967). Cavitation damage and correlations with material and fluid properties. J. Basic Eng..

[3] Paik BG (2011). Test method of cavitation erosion for marine coatings with low hardness. Ocean Eng..

[4] Im KS (2013). Unraveling the geometry dependence of in-nozzle cavitation in high-pressure injectors. Sci. Rep..

[5] Long X, Yao H, Zhao J (2009). Investigation on mechanism of critical cavitating flow in liquid jet pumps under operating limits. Int. J. Heat Mass Transf..

[6] Suryawanshi NB, Bhandari VM, Sorokhaibam LG, Ranade VV (2016). A non-catalytic deep desulphurization process using hydrodynamic cavitation. Sci. Rep..

[7] Dular M (2016). Use of hydrodynamic cavitation in (waste)water treatment. Ultrason. Sonochem..

[8] Saharan VK, Pandit AB, Kumar PS, Anandan S (2012). Hydrodynamic cavitation as an advanced oxidation technique for the degradation of acid red 88 dye. Ind. Eng. Chem. Res..

[9] Liao A, Hung C, Chen H, Chiang C (2018). Ultrasound-mediated edf-coated-microbubble cavitation in dressings for wound-healing applications. Sci. Rep..

[10] Andrej S, Tadej S, Martin P, Matevz D (2017). The issue of cavitation number value in studies of water treatment by hydrodynamic cavitation. Ultrason.-Sonochem..

[11] Abdulaziz AM (2014). Performance and image analysis of a cavitating process in a small type venturi. Exp. Thermal Fluid Sci..

[12] Meneguzzo F (2019). Real-scale integral valorization of waste orange peel via hydrodynamic cavitation. Processes..

[13] Kjeldsen M, Arndt R, Effertz M (2000). Spectral characteristics of sheet/cloud cavitation. J. Fluids Eng. Trans. Asme..

[14] Mostafa N, Karim MM, Sarker MM (2016). Numerical prediction of unsteady behavior of cavitating flow on hydrofoils using bubble dynamics cavitation model. J. Appl. Fluid Mech..

[15] Sato K, Hachino K, Saito Y (2004). Inception and dynamics of traveling-bubble-type cavitation in a venturi. Trans. Japan Soc. Mech. Eng. B..

[16] Sayyaadi H (2010). Instability of the cavitating flow in a venturi reactor. Fluid Dyn. Res..

[17] Long XP (2017). Experimental investigation of the global cavitation dynamic behavior in a venturi tube with special emphasis on the cavity length variation. Int. J. Multiph. Flow.

[18] Jahangir S, Hogendoorn W, Poelma C (2018). Dynamics of partial cavitation in an axisymmetric converging-diverging nozzle. Int. J. Multiph. Flow.

[19] Leroux JB, Astolfi JA, Billard JY (2004). An experimental study of unsteady partial cavitation. J. Fluids Eng..

[20] Furness RA, Hutton SP (1975). Experimental and theoretical studies of two-dimensional fixed-type cavities. J. Fluids Eng..

[21] Stutz B, Reboud JL (1997). Experiments on unsteady cavitation. Exp. Fluids.

[22] Dular M, Khlifa I, Fuzier S, Maiga MA, Coutierdelgosha V (2012). Scale effect on unsteady cloud cavitation. Exp. Fluids.

[23] Huang B, Zhao Y, Wang GY (2014). Large eddy simulation of turbulent vortex-cavitation interactions in transient sheet/cloud cavitating flows. Comput. Fluids.

[24] Ji B, Luo XW, Arndt RE, Peng XX, Wu YL (2015). Large eddy simulation and theoretical investigations of the transient cavitating vortical flow structure around a NACA66 hydrofoil. Int. J. Multiph. Flow.

[25] Gopalan S, Katz J (2000). Flow structure and modeling issues in the closure region of attached cavitation. Phys. Fluids.

[26] Chen GH (2015). Combined experimental and computational investigation of cavitation evolution and excited pressure fluctuation in a convergent-divergent channel. Int. J. Multiph. Flow.

[27] Chen GH, Wang JY, Hu CL, Huang B, Zhang MD (2015). Observations and measurements on unsteady cavitating flows using a simultaneous sampling approach. Exp. Fluids.

[28] Charriere B, Goncalves E (2017). Numerical investigation of periodic cavitation shedding in a venturi. Int. J. Heat Fluid Flow.

[29] Kuldeep K, Saharan VK (2016). Computational study of different venturi and orifice type hydrodynamic cavitating devices. J. Hydrodyn..

[30] Ghassemi H, Fasih HF (2011). Application of small size cavitating venturi as flow controller and flow meter. Flow Meas. Instrum..

[31] Gogate PR (2011). Hydrodynamic Cavitation for Food and Water Processing. Food Bioprocess Technol..

[32] Tian H, Zeng B, Yu NJ, Cai GB (2014). Application of variable area cavitating venturi as a dynamic flow controller. Flow Meas. Instrum..

[33] Cai J, Huai X, Li X (2009). Dynamic behaviors of cavitation bubble for the steady cavitating flow. J. Therm. Sci..

[34] Ganesh H, Makiharju SA, Ceccio SL (2016). Bubbly shock propagation as a mechanism for sheet-to-cloud transition of partial cavities. J. Fluid Mech..

[35] Stutz B, Legoupil S (2003). X-ray measurements within unsteady cavitation. Exp. Fluids.

[36] Pawar SK (2017). Sonochemical effect induced by hydrodynamic cavitation: Comparison of venturi/orifice flow geometries. AIChE J..

[37] Aeschlimann V, Prothin S, Barre S, Djeridi H (2012). High speed visualizations of the cavitating vortices of 2D mixing layer. Eur. J. Mech. B/Fluids..

[38] Kumar P, Chatterjee D, Bakshi S (2017). Experimental investigation of cavitating structures in the near wake of a cylinder. Int. J. Multiph. Flow.

